# Development of a Cost-Effective Pediatric Intubation Task Trainer for Rural Medical Education

**DOI:** 10.7759/cureus.6604

**Published:** 2020-01-08

**Authors:** Stuti Tanya, Adam Dubrowski

**Affiliations:** 1 Medical Education and Simulation, Memorial University of Newfoundland, St. John's, CAN; 2 Health Sciences, Ontario Tech University, Oshawa, CAN

**Keywords:** pediatric, intubation, airway management, training, simulation, task-trainer, medical education, 3d printing, anaesthesia, emergency medicine

## Abstract

Pediatric intubation and airway management (PIAM) is a life-saving, emergent procedure that is performed by a variety of healthcare practitioners. Securing the pediatric airway in a time-sensitive fashion is a specialized skill that declines with lack of practice, leading to a precarious gap in clinical competency and healthcare delivery. However, current training models for PIAM, such as live animals, human cadavers, and simulators, are not adequately accessible or reliable due to their combination of high cost, unrealistic simulation, lack of standardization, and ethical concerns. Task trainers pose an ethically and fiscally sustainable training model for experiential learning through repetitive practice, which has been shown to dramatically improve trainee proficiency and confidence in performing high-acuity low-occurrence procedures such as pediatric intubation. This work aims to report the development process and initial validation evidence of a prototype cost-effective pediatric intubation task trainer that can be used for post-graduate education, especially in resource-challenged settings.

## Introduction

Medical education is often described as drinking from a firehose, and rightly so: trainees acquire an onslaught of knowledge and skills in a very short period of time, with the expectation that they will be able to proficiently exercise these skills upon demand. However, research shows that much of this expertise fades with lack of practice, especially in regard to high-acuity low-occurrence (HALO) skills such as pediatric intubation and airway management (PIAM) [1]. Pediatric patients present with greater difficulty in airway management due to anatomical differences and varying pathological processes compared with their adult counterparts, for whom practitioners are trained [2]. This challenge is further exacerbated in practitioners who are not regularly exposed to manipulation of the pediatric airway-much less in the crisis situations in which intubation is often required-leading to marked deficits in psychomotor skill retention [1,3]. Inability to secure the pediatric airway in a time-sensitive fashion causes suboptimal success rates in this life-saving, emergent procedure [4].

 Adequate training models for PIAM are in dire need, particularly in global and rural contexts, due to the high prevalence of scenarios that require airway management without specialized personnel such as anesthesiologists, emergency physicians, and respiratory therapists [5-7]. There are three general classifications of models used for teaching PIAM: live animals, human cadavers, and simulators. Attempts to use live animals or human cadavers have shown promise for experiential learning of pediatric intubation but pose ethical concerns, can be difficult to acquire, and are not available for long-term practice [8]. These limitations are further amplified in both global and rural contexts, where availability and expenditure are limited. The shortfalls of both live and cadaveric models comprise the strengths of simulators. Emerging evidence suggests that experiential learning with simulation models improves intubation performance in medical trainees, while being ethically sound and offering limitless opportunity for practice. Still, current simulation models only offer moderate realism and can be very expensive, lending to financial and geographical barriers to accessibility. The demand for a cost-effective pediatric intubation training model with reasonable realism prevails, and the solution may lie in developing three-dimensional (3D)-printed models, which hold promise for improving trainee confidence when paired with repetitive, problem-based experiential learning [9-10].

Task trainers exist within the realm of simulation models, but they attempt to train a particular task through repetition rather than simulate the reality of a clinical experience, as with a simulation model. As such, task trainers adopt a minimalist “partial-body” design in contrast to simulation models and are therefore significantly more cost-effective [11-12]. Simulation models have come to represent a luxury in medical education, but task trainers are equipped only with necessary features designed to train basic skills and improve trainee confidence, particularly with respect to HALO skills, which benefit exponentially from any practice due to the infrequency of exposure to real clinical scenarios [13]. Although task trainers do not facilitate an equivalent degree of realism as simulation models, such as real-time physiological feedback, they do offer potential to be used imaginatively with case-based learning scenarios, thus further widening their scope of training utility. Developing a 3D-printed task trainer has a far-reaching impact on both resource-rich and resource-challenged areas, which can benefit immensely from simulation-based health professional education [14]. Here, our objective is to develop a prototype PIAM task trainer to assess demand and critical features that are necessary for producing a high-quality model. Ultimately, the goal of the simulation community would be to develop a design for the task trainer that can be printed anywhere in the world for a miniscule fraction of the cost of a simulation model.

## Technical report

This technical report is organized into five sections: design considerations and education context, design protocol, development, evaluation methods, and evaluation results.

Design considerations and education context 

The need for a PIAM task trainer stems primarily from the anatomical differences of neonate and pediatric patients in comparison with adults, on whom much of medical education is centered. In fact, the Mallampati classification system, which is based on visibility of the glottis, fails to accurately predict the ease of intubation in pediatric patients due to these anatomical differences [15]. The larger head and prominent occiput of pediatric patients predispose them to airway obstruction, especially when asleep, and poses difficulty in correctly aligning the airway during PIAM. Pediatric patients also have a proportionally smaller mandible but a larger tongue, shorter and narrower hypopharynx, higher larynx and cricoid ring, and prominent adenoids and tonsils, resulting in insufficient upper airway space. Decreased tone of upper airway muscles upon administration of anesthetic or sedative drugs introduces further challenges to securing the pediatric airway, and patients may be more susceptible to trauma of the vocal chords due to their obtuse alignment. Prior studies attest that the pediatric airway is funnel-shaped, with the narrowest portion most superior at the elliptical cricoid ring, whereas the adult airway is more cylindrical, and narrowest at the level of the glottis. Physiological differences in oxygen consumption, residual lung capacity, and respiratory rate in pediatric patients reduce the time available to perform the procedure successfully. These differences are most pronounced upon birth and gradually disappear as the child grows older [2].

The purpose of simulation-based medical education in pediatrics is to facilitate a “learning event with goals and objectives” that is not “a replacement for clinical experiences” but rather “a safe place to learn … without the fear of harming patients” [11]. In traditional clinically oriented medical education, the concept of “psychological safety” of learners is underemphasized yet crucial to establishing proficiency and confidence in essential skills [11]. Simulators act as a mimetic patient, often equipped with advanced technology to reproduce realistic physiological responses. As such, they are often tremendously high realism, but also high cost and high maintenance. This makes simulators an investment to own, upkeep, and replace, especially when used to educate masses of students who require repetitive practice. Task trainers, on the other hand, are not designed to simulate a whole-body patient. Instead, their purpose is to offer a mode for training psychomotor skills for isolated tasks such as pediatric intubation and surgical airway management. They do not feature computerized controls or lifelike aesthetics but are engineered to provide the absolute essentials in facilitating a sufficiently realistic experience, such as accurately replicated anatomy and texture [11]. Thus, they are significantly cheaper to produce and maintain: the cost of owning and operating a 3D printer, plus the materials to produce a task trainer, would average at less than half the cost of a simulation model [1,16].

In the case of a PIAM task trainer, realism would be directly associated with cost; therefore, a variable cost-effective task trainer could be feasibly developed for availing consumers from different economic conditions. Essentially, we planned to develop a base-level trainer with upgradable options to increase realism or flexibility of scenarios. This would increase accessibility of the trainer to resource-challenged areas, in which trainees may opt to purchase the base-level trainer for initial practice and then upgrade over time if the fiscal opportunity arises. It would also allow for a simple and an inexpensive replacement of parts if needed. For example, the base-level trainer would comprise the essential structure of the model composed of realistically textured materials to simulate the oral cavity, neck, and jaw flexibility for head and neck tilt, and cricoid cartilage, leading into lung and stomach balloons to confirm correct tube placement. It would function to simulate the manual task of pediatric intubation with no physiological feedback or complications. The consumer may then wish to add replenishable reservoirs for artificial bodily fluids to mimic saliva or vomit, inflatable tongue to simulate tongue edema or pharyngeal swelling, and inflatable trachea to simulate variable airway resistance or lung compliance, among other potential add-ons. These optional upgrades, which are inspired by components of high-realism models presently in the Canadian market, would simulate pediatric intubation with positive feedback, thus introducing the trainee to a more difficult, multifaceted airway management scenario that more closely mimics reality [17]. With further technological developments, it may be possible to hybridize rudimentary task trainers with virtual reality simulators that overlay a paradoxically nontangible realism to the procedural skill training experience, again at a fraction of the cost of whole-body simulators [11].

Design protocol

**Figure 1:**
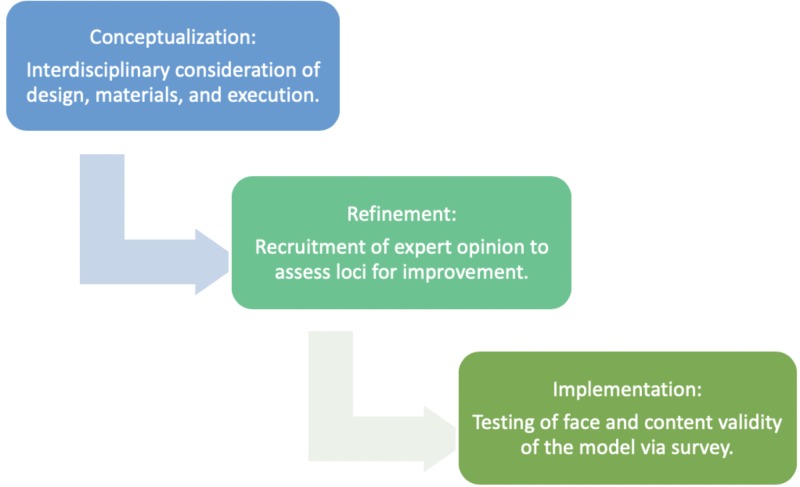
Three-phase design process to develop the prototype task trainer

The three-phase-“conceptualization, concept refinement, and implementation”-process to develop our prototype was guided with expert opinion from a pediatric anesthesiologist [18]. We developed an initial prototype by extracting anatomical features from a computed tomography (CT) scan of a five-year-old patient with normal anatomy, custom 3D computer-aided design modeled components, and pre-existing models from open source websites for review and practice. Opinions obtained from our first task trainer were used to inform the development of our second and final iteration, which then was further reviewed by a cohort of pediatric anesthesiologists to assess the trainer’s face and content validity. Since we were working in a resource-challenged setting, texture and color accuracy of our materials were not possible for some components of the model. Anatomical accuracy of materials with respect to texture, color, flexibility, and movement was prioritized, and any areas for improvement were incorporated into the second iteration of our trainer. Our design process is visually represented in Figure 1.

Development

Base Board

Using Fusion 360 (AutoDesk, San Rafael, CA, USA), the base of the task trainer was designed to represent the top portion of the body that houses the airway. A guided slot was incorporated to allow for the head tilt feature of the skull, and a rounded extrusion simulated the shoulder girdle with an angled opening for the guidance of the airway during the simulation.

We used the Ultimaker S5 3D printer (Ultimaker, Utrecht, the Netherlands) for printing and molds for casting. A dual-extrusion print with black polylactic acid (PLA) 2.85-mm filament was used for the base with InnoSolve 2.85-mm polyvinyl alcohol (PVA) support filament (InnoSolve, Castle Rock, CO). A layer height of 0.2 mm, infill of 10%, support material infill of 15%, and print speed of 70 mm/s at a temperature of 205°C was used. The print instructions file was saved to a secure USB drive to upload to the 3D printer.

**Figure 2:**
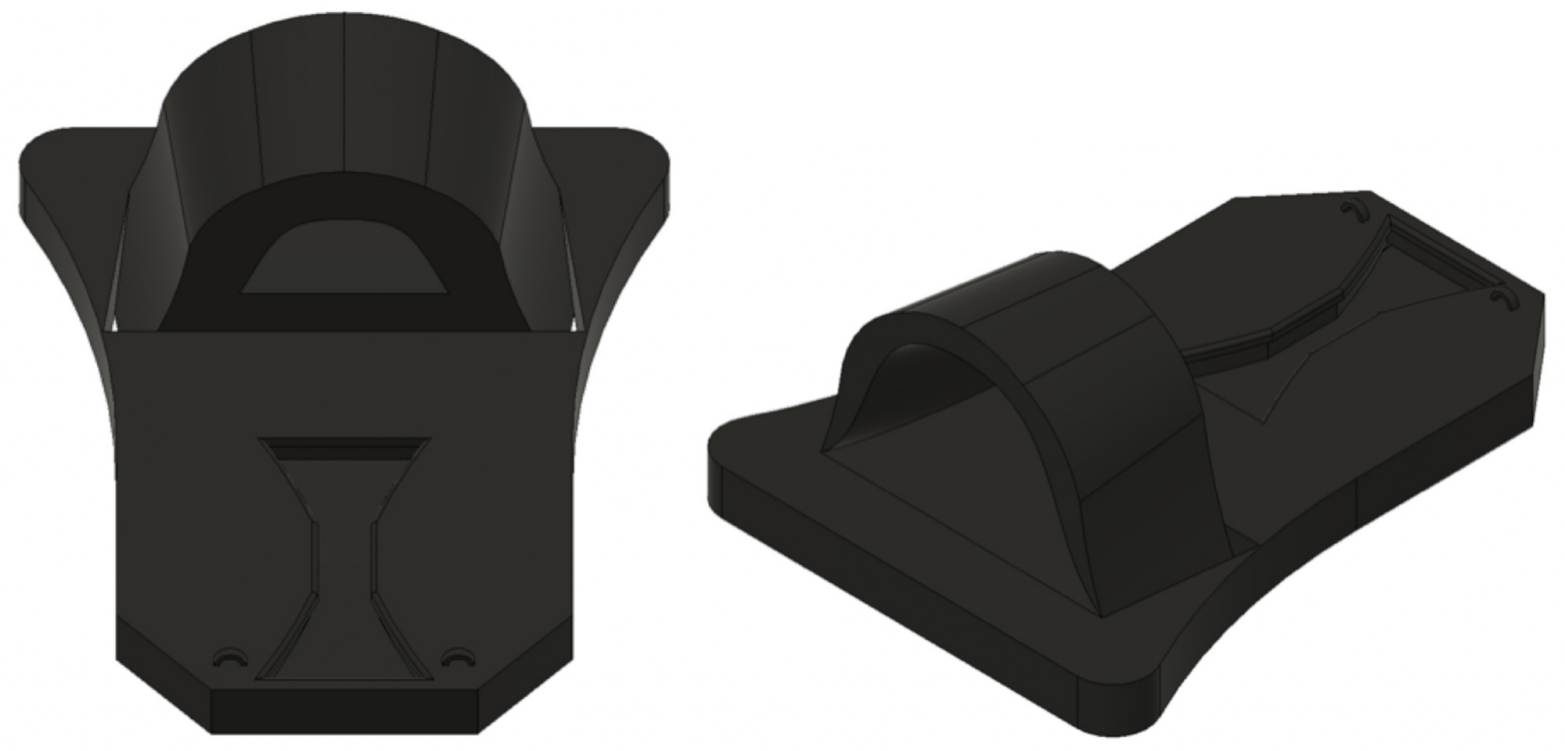
Base board

Upon review from our clinical advisor, the primary recommendation was to remove the thoracic cavity casing as it was interfering with head-tilt and jaw movement. We also moved the thoracic cavity closer to the guided head slot and angled the airway bed slightly upward to facilitate the airway in its proper axis during intubation since the positioning in the first iteration limited the physiological range of motion. Furthermore, since the pediatric occiput offers resistance upon tilting during PIAM, we also made the occiput attachment loops slightly thicker to prevent the loops from breaking when tilting the head, ensuring longevity of the task trainer. The base board is depicted in Figure 2.

Occiput

The CT scan captured anatomy from the eye level of the patient to the mid-chest level; therefore, complete extraction of the skull was not possible. As the superior occiput does not serve a vital function in this simulation, a pre-existing model of an adult skull from an open-source royalty-free website (thingiverse.com) was downloaded and rendered using a combination of MeshMixer (AutoDesk) and Fusion 360. The occiput was scaled down appropriately using measurements taken from the CT scan in OsiriX MD (Pixmeo SARL, Bernex, Switzerland). For purposes of the trainer, an attachment for the bottom jaw was added in Fusion 360, consisting of two pins that extended at the attachment site and a ridge that would prevent the jaw from opening wider than anatomically possible. To simulate the head tilt that positions the child’s occiput correctly for the procedure, a curved guide was attached to the occipital protuberance that could glide on the base of the task trainer. Using Fusion 360, the model was then converted to an STL (STereoLithography) file for 3D printing purposes.

The Ultimaker 3 3D printer was used to develop the occiput with a dual extrusion print. The body of the skull was printed using PLA filament material in ivory color to more closely resemble bone, with PVA acting as support material. A total of 338 g of PLA filament and 177 g of PVA filament were used, with a total print time of 2 days 6 hours and 26 minutes. The recommended settings were adjusted to a layer height of 0.2 mm, infill of 10%, support material infill of 15%, and print speed of 70 mm/s at a temperature of 205°C.

**Figure 3:**
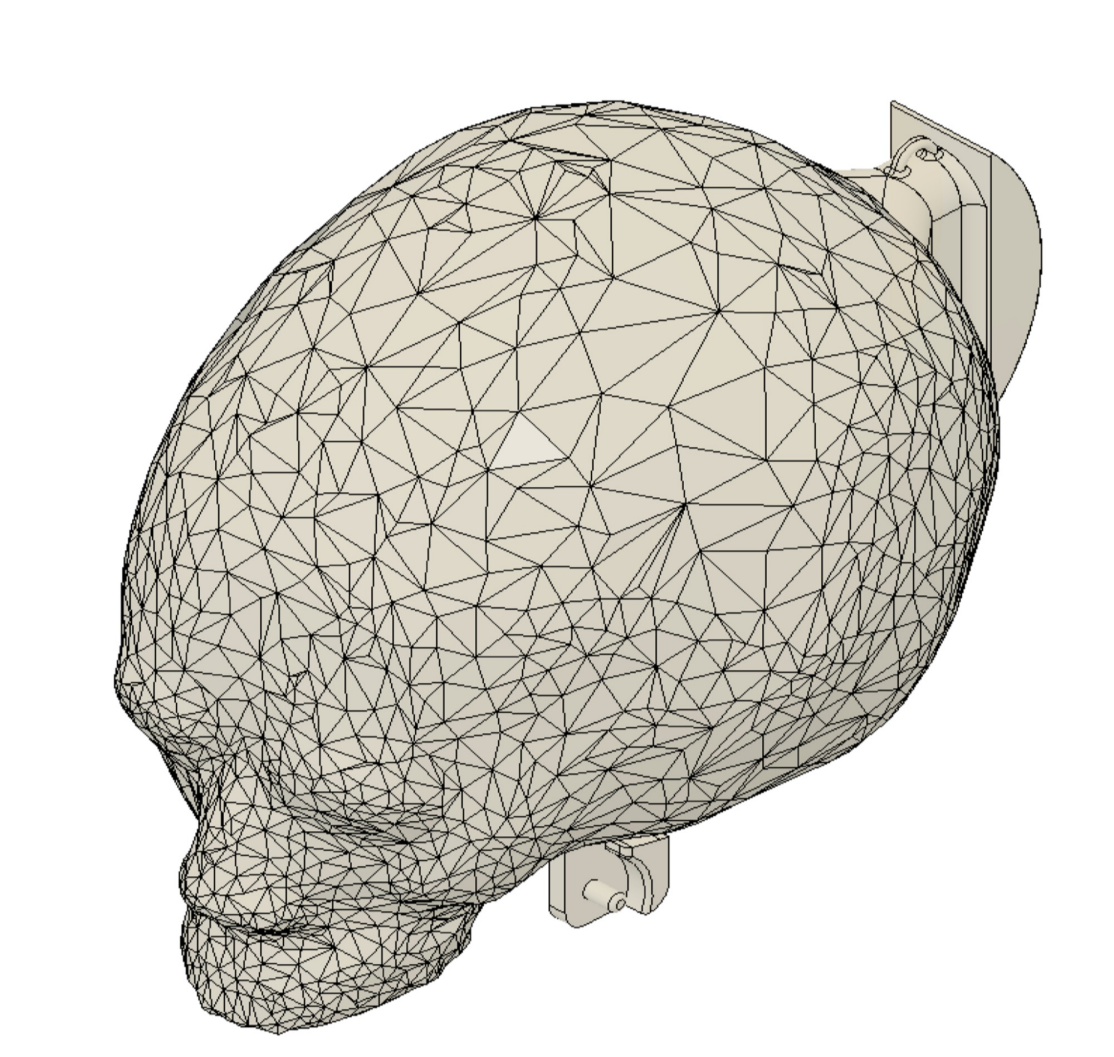
Occiput

Upon review from our clinical advisor, the skull was deemed appropriate and did not require any adjustments. The occiput is depicted in Figure 3.

Jaw

The bottom jaw model was extracted from the provided CT scan with the use of OsiriX MD. OsiriX MD sliced the CT scan into 0.8-mm layers that could be easily viewed to identify the different anatomical features of interest. With 3D volume rendering and two-dimensional (2D) surface rendering, the jaw structure was extracted from the scan and exported as an STL file. The jaw was inserted into Fusion 360 as a mesh and altered to provide attachment sites to the upper jaw and mouth cavity. This file was then imported into MeshMixer to refine and smooth the model using a variety of sculpting tools. The final design was exported as an STL for 3D printing.

As with the occiput, the Ultimaker 3 3D printer was used to print and cast the jaw in a dual-extrusion print. The jaw was constructed similarly to the occiput, with ivory-colored PLA filament, with PVA filament as support. A total of 25 g of PLA filament and 10 g of PVA filament were used, with a total print time of 3 hours 52 minutes. The following recommended settings were used: a layer height of 0.2 mm, infill of 20%, support material infill of 15%, print speed of 70 mm/s at a temperature of 205°C.

**Figure 4:**
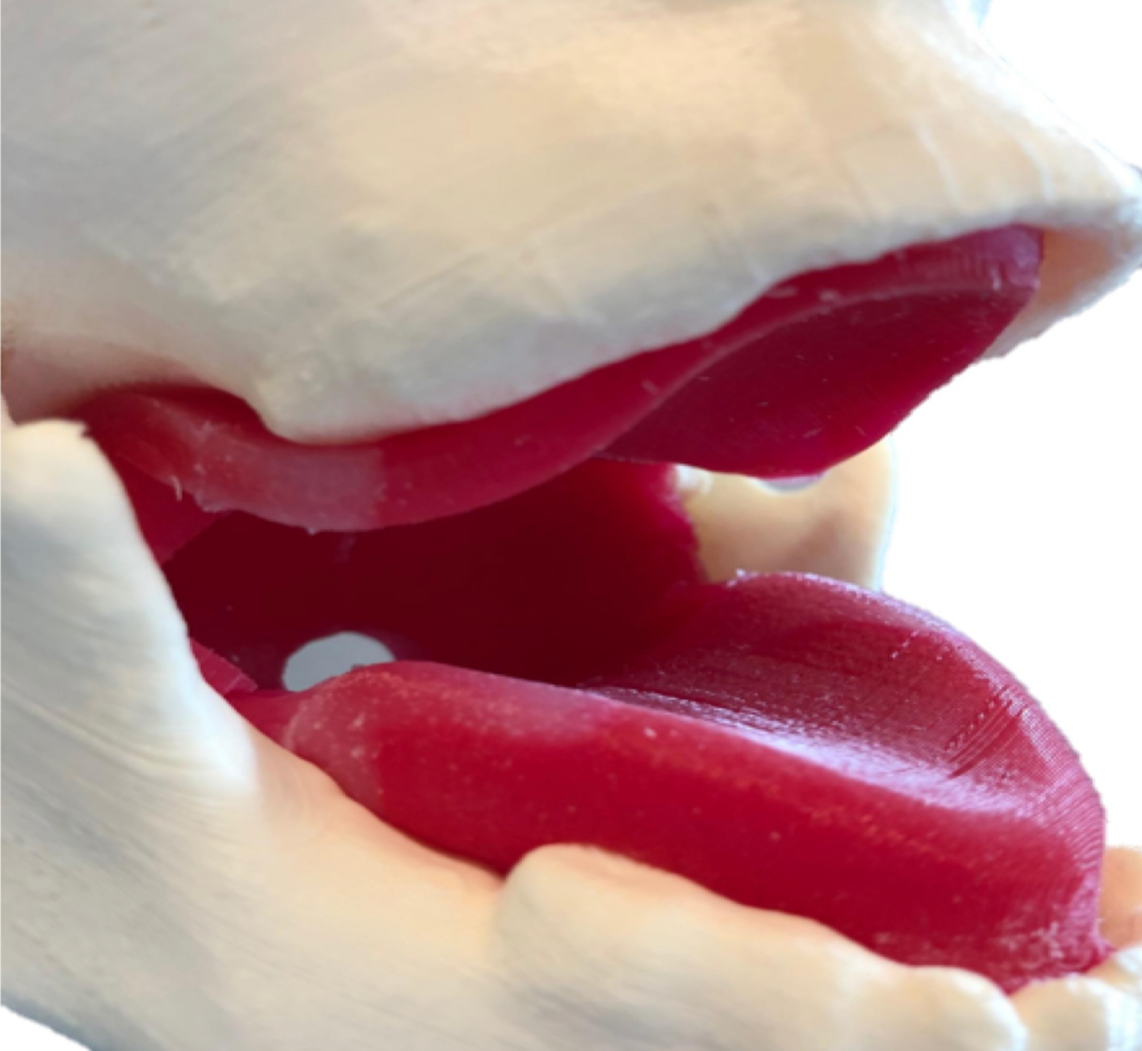
Jaw and oral cavity

Upon review from our clinical advisor, we added angular restrictions on the attachment pegs to the jaw to prevent the mouth from opening further than anatomically possible. This restriction helps to facilitate what may be deemed one of the most important features of PIAM: the smaller oral cavity, which makes maneuvering and visualization more difficult in pediatric patients than their adult counterparts. The jaw along with the oral cavity is depicted in Figure 4.

Oral Cavity

The oral cavity comprises silicone in which a 3D-printed mold was required. The oral cavity was designed in Fusion 360 with reference to the size and shape of the upper and lower jaw. The bottom was thickened to simulate the tongue, and the top as the roof of the child’s mouth. An opening for insertion of the airway was formed at the back of the mouth. To cast the feature, a three-part mold was modeled in Fusion 360. The model of the mouth previously created was imported into the design and combined into the mold in such a way that the element was cut away from the mold leaving the oral cavity negative imprinted in the mold to be filled with silicone. The oral cavity along with the lower jaw is depicted in Figure 4.

The Ultimaker 3 3D printer was used for the mouth cavity as well, with a dual-extrusion print. The mold of the mouth was printed in PLA, with PVA as support. A total of 101 g of PLA filament and 8 g of PVA filament were used, with a total print time of 8 hours 34 minutes. The settings used are as follows: layer height of 0.2 mm, infill density of 20%, support material infill of 15%, and print speed of 70 mm/s at a temperature of 205°C. Using the mold, the oral cavity was cast using silicone. The silicone mix used was Ecoflex 00-30 (Smooth-On, East Texas, PA, USA) platinum cure silicone rubber (part A and part B in a 1:1 ratio) measuring 100 mL in total. Parts A and B were combined by mixing, and a small amount of red pigment was added to add an element of realism to the mouth. The mixture was then poured into the assembled three-part mold and left to cure for a total of 4 hours.

Upon review from our clinical advisor, we increased the thickness of the bottom of the mouth cavity to simulate the tongue. In the first iteration, we aspired to create a hollow inflatable tongue to simulate tongue edema, which may occur in PIAM scenarios. However, the inflatable tongue posed various limitations and reduced overall realism; therefore, it was ultimately removed from the second iteration of our prototype. This modification allows for the tongue and mouth cavity to be printed as a singular piece, eliminating the need for attachment. The first iteration also included removable teeth to simulate dental injury during intubation, which may be more common in pediatric scenarios due to the smaller oral cavity. However, these removable teeth would fall out too easily and the underlying pegs were highly vulnerable to damage. To simplify the task trainer and reduce the necessity for extra printing and replacement of parts, we decided to replace these removable teeth with stationary teeth. It was reasoned that trainees would be aware of the possibility of dental injury through tactile contact with stationary teeth as well.

Airway

The airway and esophagus were extracted solely from the CT scan. Using OsiriX MD, a new region of interest was created to localize the airway-esophagus unit, and then 3D surface rendering was used to remove undesired portions of the scan. Then, the 2D surface rendering tool was used to further isolate the airway and export the model as an STL file. This file was imported into MeshMixer to eliminate any remaining undesired components, and we further refined the model using MeshMixer's model sculpting tools.

The Stratasys Connex3 3D printer (Stratasys, Eden Prairie, MN, USA) was used for the airway to facilitate greater precision. The model was opened using Object3D, where the desired placement and validation were performed. The airway was printed in the flexible Tango Black+ resin (Stratasys) using 146 mL of total and 197 mL of support material. Ideally, this print would have been done in a more anatomically correct pink-red color rather than the resultant dark-grey. The total print time was 7 hours 26 minutes.

**Figure 5:**
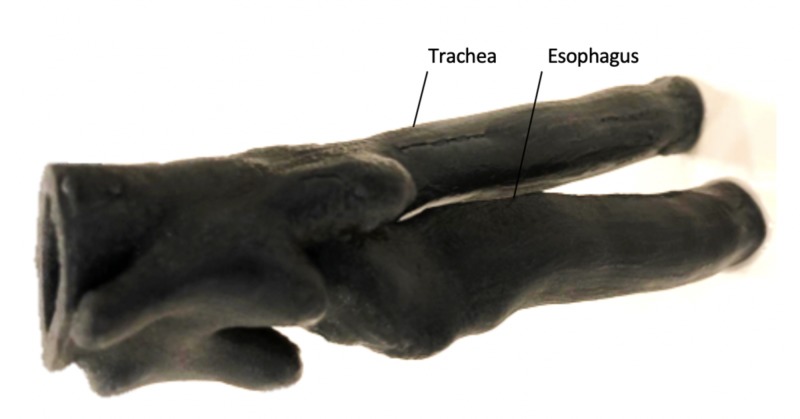
Airway

Upon review from our clinical advisor, we scaled the airway-esophagus unit up by 1.3 times for two reasons: first, the original airway was smaller than average for a five-year-old patient, and, second, lubrication is typically used in intubation, and soft muscle tissue is more elastic, which allows the endotracheal tube to be inserted with less resistance. Since our task trainer is not compatible with lubrication media due to its silicone-based construction, we decided to scale the airway up by a small factor to compensate. We also printed this unit in a more flexible material in the second iteration since the first iteration resulted in an unrealistically firm airway that cracked while practicing intubation. We considered casting the airway in firm silicone, as with the tongue, in which case a red or pink airway would have been possible to create. However, the CT scan anatomy was too intricate to replicate in a mold; therefore, a silicone model was not feasible for this iteration. Both the airway and the esophagus terminate in color-coded balloons: inflation of the pink balloon after intubation would indicate correct placement in the trachea and subsequent perfusion of the lungs, whereas inflation of the blue balloon after intubation would indicate misplacement in the esophagus and subsequent perfusion of the stomach. The airway is depicted in Figure 5. 

**Figure 6:**
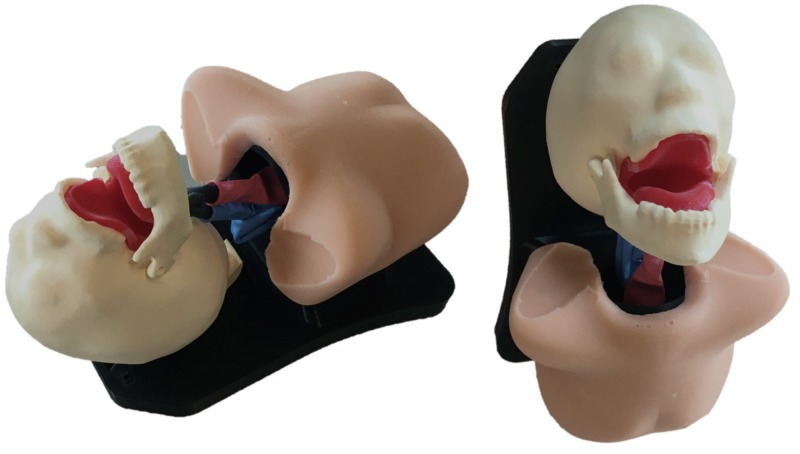
Final iteration of the task trainer

The second and final iteration of the task trainer is depicted in Figure 6.

Evaluation methods 

The task trainer was evaluated by five pediatric anesthesiologists with 4 to 20 years of experience who regularly perform pediatric intubation in a variety of settings including the operating room, emergency room, and intensive care unit. Their opinions were gathered through a semi-structured interview with guided questions to provide evidence for face and content validity, in other words, realism and efficacy of the task trainer as a tool for medical education. Participants were given a laryngoscope, endotracheal tube, stylet, and a bag valve mask during their interaction with the task trainer. After their interaction with the model, the following metrics were evaluated: physical attributes, realism of experience, and efficacy of the task trainer as a tool for medical education, as well as general comments and suggestions for improvement. The answers were audio-recorded and transcribed offline by a single researcher.

Evaluation results

The interview findings are paraphrased below with specific quotes from participants.

All participants unanimously emphasized the potential of our PIAM task trainer as an innovative tool to fill the dire gap in PIAM training. The “customizability” of the task trainer and its potential for application in resource-challenged settings were acclaimed as its strongest features. One participant commented: “the airway material is more pliable than other task trainers, which is a positive feature. Although a new pediatric intubation trainer is definitely necessary, improvements in this model are required”. Many other participants shared the following feedback: despite the task trainer being “a positive step”, there were several loci for improvements and it was ultimately deemed in need of significant improvement prior to consideration for practical use in medical education. This feedback was consistent across the participants’ comments.

Tissue color for airway structures was universally touted as the main disadvantage of the trainer: the black color made illumination with the laryngoscope difficult due to light absorption such that vital landmarks in intubation, including glottic structures and vocal chords, could not be clearly identified. Nearly all participants commented on color, with one participant saying: “The color of the airway must be changed to flesh color. The black material absorbs the light”. Access to the vallecula and the interface between the oral cavity and pharynx were condemned for being unrealistic due to “rigidity of the airway material”. All articulations in the model were considered to be in need of reinforcement, particularly the skull-base board and oral cavity-pharynx interfaces, as these would "become easily disconnected, causing the model to collapse".

The universal comments on color and articulations point to their paramount importance in facilitating a realistic simulation experience. Although the model enjoyed an optimistic outlook among its criticisms, one evaluator identified the dangers in such task trainers that are critical to note in the development of future simulation models. Their concern was that very few trainers are truly representative of anatomy: "by reproducing unrealistic scenarios, and having junior learners succeed, we may be giving them a false sense of security for the real-life event”. Such comments went beyond the scope of the interview, which was to address the realism and education value of the task trainer, but are nonetheless important in ensuring a holistic and contextual understanding simulation and medical education [19].

## Discussion

Use of 3D printing for medical education is a relatively new phenomenon rapidly gaining attention, and pediatric intubation occupies a small niche in the world of simulation model development. We sought to reduce barriers in PIAM training by gauging interest in a cost-effective, ethically sound 3D-printed task trainer. The primary objective of this rudimentary task trainer is accessibility for trainees around the world, even in resource-challenged areas. As this task trainer is the first of its kind in PIAM, our design and development process anticipated a rudimentary prototype model that would assess demand and interest from experienced professionals.

Perhaps, the most imperative criticism we received not only for our task trainer but also for all simulation models is a necessity for unparalleled realism. Realism cannot be compromised if the task trainer is to be an effective learning tool, even when building a task trainer that attempts to offer more value, dollar-for-dollar, than simulators. Numerous studies have demonstrated the efficacy of task trainers in improving clinical proficiency by providing a tool for experiential learning; still, pediatric simulators and task trainers have yet to break the mainstream market [11]. This may be due to the complexity of the anatomy and the onus of the practitioner to perform the procedure with minimal, if any, error during real PIAM scenarios. Indeed, the procedure is considered a HALO skill with a minimal margin of error. It is important to develop an adequate task trainer for PIAM; after all, “children are not merely small adults” and the management of pediatric scenarios requires a specialized skill set that can only be acquired with repetitive, procedural skill training [2].

Some avenues for improvement, besides working with anatomically correct materials and colors, include collecting additional CT scans to compile a more anatomically accurate representation of the pediatric airway for 3D modelling. MRI imaging of the pediatric airway, although uncommon, would be beneficial for elucidating density and texture of the vital soft tissue structures that are abundant in the airway. With enhancements in material color, texture, and overall structural robustness, a truly efficacious task trainer is within the realm of possibility for 3D-printed simulation modelling. It is in this capacity that task trainers demonstrate their full potential in being an equalizing force in the healthcare arena, by opening access to even the most resource-challenged communities, practitioners all over the world can become proficient in versatile, life-saving procedures such as PIAM.

## Conclusions

We built a relatively inexpensive and standardized PIAM task trainer, primarily for use in resource-challenged settings. Our prototype demonstrates initial agreement between experts in that the task trainer is necessary given the lack of simulation models for PIAM; however, improvements in realism are required before implementing the task trainer for medical education purposes. To be truly effective, this task trainer and similar simulators need to be implemented in conjunction with effective scenarios and execution of the scenario supported by educational theory, and, finally, the task trainer must fit with the needs of its trainees. Future work will focus on refining the model and developing case scenarios for PIAM that can simulate the entirety of the clinical PIAM experience.
